# Preparation of COPs Mixed Matrix Membrane for Sensitive Determination of Six Sulfonamides in Human Urine

**DOI:** 10.3390/molecules28217336

**Published:** 2023-10-30

**Authors:** Ying Liu, Yong Zhang, Jing Wang, Kexin Wang, Shuming Gao, Ruiqi Cui, Fubin Liu, Guihua Gao

**Affiliations:** 1 School of Pharmacy, Jining Medical University, Rizhao 276826, China; ying1737883492@126.com (Y.L.); zhang_2211@163.com (Y.Z.); tawkx589@163.com (K.W.); 17860202743@163.com (S.G.); 13697839528@163.com (R.C.); liufblvy@163.com (F.L.); 2 School of Pharmacy, Shandong University of Traditional Chinese Medicine, Jinan 250355, China; wangjing20210903@163.com

**Keywords:** covalent organic polymer, mixed matrix membranes, TpDMB-COPs, membrane solid-phase extraction, HPLC, sulfonamides

## Abstract

In this study, TpDMB-COPs, a specific class of covalent organic polymers (COPs), was synthesized using Schiff-base chemistry and incorporated into a polyvinylidene fluoride (PVDF) polymer for the first time to prepare COPs mixed matrix membranes (TpDMB-COPs-MMM). A membrane solid-phase extraction (ME) method based on the TpDMB-COPs-MMM was developed to extract trace levels of six sulfonamides from human urine identified by high-performance liquid chromatography (HPLC). The key factors affecting the extraction efficiency were investigated. Under the optimum conditions, the proposed method demonstrated an excellent linear relationship in the range of 3.5–25 ng/mL (*r*^2^ ≥ 0.9991), with the low limits of detection (LOD) between 1.25 ng/mL and 2.50 ng/mL and the limit of quantification (LOQ) between 3.50 ng/mL and 7.00 ng/mL. Intra-day and inter-day accuracies were below 5.0%. The method’s accuracy was assessed by recovery experiments using human urine spiked at three levels (7–14 ng/mL, 10–15 ng/mL, and 16–20 ng/mL). The recoveries ranged from 87.4 to 112.2% with relative standard deviations (RSD) ≤ 8.7%, confirming the applicability of the proposed method. The developed ME method based on TpDMB-COPs-MMM offered advantages, including simple operation, superior extraction affinity, excellent recycling performance, and easy removal and separation from the solution. The prepared TpDMB-COPs-MMM was demonstrated to be a promising adsorbent for ME in the pre-concentration of trace organic compounds from complex matrices, expanding the application of COPs and providing references for other porous materials in sample pre-treatment.

## 1. Introduction

Sulfonamides (SAs) have been widely used to prevent bacterial infectious diseases in food-borne animals due to their efficacy, stability, and affordability [1]. However, excessive and prolonged use of SAs has led to residues in animal products, which can be ingested by humans through the food chain [2,3]. This may result in various toxic side effects, such as allergic reactions, drug resistance, and teratogenicity, as well as carcinogenic and mutagenic effects. Approximately ninety percent of SAs and their metabolites are excreted in human and animal urine after application due to incomplete absorption and metabolism [4,5]. As urine is the primary excretory pathway for SAs, its content can indirectly indicate the degree of human contamination SAs [6,7]. Numerous countries and organizations have already restricted or prohibited the use of SAs. Therefore, there is a pressing demand for an efficient, rapid, and accurate method to analyze SAs in human urine.

Modern analytical technologies, including high-performance liquid chromatography (HPLC) [8,9], fluorescence detection [10], capillary electrophoresis with ultraviolet detection (CE-UV) [11,12], and liquid chromatograph-tandem mass spectrometer (LC–MS/MS) [13,14,15,16], have been used to determine SAs drugs in recent decades. Among them, HPLC is the most commonly employed method for the determination of SAs, due to its sensitivity, specificity, and ability to identify unknown substances. However, direct measurement of low SAs concentrations in complex human urine is challenging [17]. Sample pre-treatment is a crucial step in the analytical process due to the low concentrations of SAs and complex matrix in human urine. Various methods have been utilized to pre-treat SAs residues in different matrices, such as liquid-liquid extraction [18,19], solid-phase extraction [20], and solid-phase microextraction [21]. Classical extraction methods, such as solid-phase extraction (SPE) and dispersive solid-phase extraction (dSPE), are commonly preferred, while certain drawbacks, including tedious and time-consuming procedures, high operational costs, and significant solvent consumption, cannot be ignored. Therefore, a suitable and efficient sample pre-treatment is necessary. Mixed-matrix membrane solid-phase extraction (ME) [22] has garnered significant attention as an interesting method in recent years. It involves incorporating the adsorbent into the polymer matrix and allowing the mixed matrix membrane (MMM) to tumble freely through the sample under vortex force to complete the extraction of the target analytes. This approach avoids several issues, such as centrifugation for solid-liquid separation and material loss, while offering the advantages of good reproducibility, simple operation, and easy acquisition. The key component of ME is the adsorbent, and it exhibits broad applicability in the separation and purification of samples due to the variety of available adsorbents. Adsorbents with excellent performance can ensure effective enrichment and purification of samples. Various studies have explored adsorbents, such as carbon nanotubes [23], activated carbon [24], metallic organic frameworks [25], covalent organic polymers (COPs) [26], and others.

COPs, an emerging class of porous material, have gained significant attention owing to their mild synthesis conditions, excellent thermal stability, porosity, high specific surface area, and moderate to high chemical stability [27]. In general, COPs are prepared with monomers of light elements. They are constructed using covalent bonds, such as C=C, C-C, and C=N, and can be categorized as crystalline covalent organic frameworks (COFs) and amorphous COPs [28]. Although crystalline COFs are challenging to synthesize due to harsh reaction conditions, amorphous COPs can be easily prepared through a simple synthetic process. They have been widely applied in catalysis [29], gas absorption [30], sample pretreatment [31], and drug delivery [32]. The existence of carbon, oxygen, and nitrogen atoms in COP blocks enhances their enrichment efficiency as the adsorbents, owing to the inter-molecular forces with target compounds [33]. Therefore, COPs hold excellent potential for sample pretreatment. Ma et al. [34] developed a novel covalent magnetic sulfonated organic polymer for solid-phase magnetic extraction of protoberberine alkaloids. Wei et al. [35] synthesized an imine-based porous 3D COP, which proved an effective solid-phase sorbent for detecting amphenicols in water samples. These findings have demonstrated the practical and promising applications of COPs in sample pretreatment [36]. However, the use of COPs as mixed matrix membrane extraction adsorbents has seldom been explored. Previous reports have indicated that amorphous COPs are typically synthesized by polymerizing designed organic monomers, including various aldehydes and amides, through the Schiff-base reaction [37].

In this study, a COP named TpDMB-COPs was synthesized through a Schiff-base reaction using 2,4,6-triformylphloroglucinol (Tp) and 3,3′-dimethylbenzidine (DMB) as organic monomers. Subsequently, a TpDMB-COPs mixed matrix membrane (TpDMB-COPs-MMM) was prepared by embedding the TpDMB-COPs into polyvinylidene fluoride (PVDF). Based on the obtained TpDMB-COPs-MMM, a membrane solid-phase extraction method (ME) for the quantitative extraction of SAs from human urine was developed. The SAs analyzed in this method included sulfamerazine (SMR), sulfamethoxypyridazine (SMP), sulfamethizole (SMX), sulfadimethoxine (SDM), sulfadoxine (SDX), and sulfaquinoxaline (SQ), which were determined by HPLC.

## 2. Results and Discussion

### 2.1. Characterization of Tp-DMB-COPs and Tp-DMB-COPs-MMM

**Figure 1:**
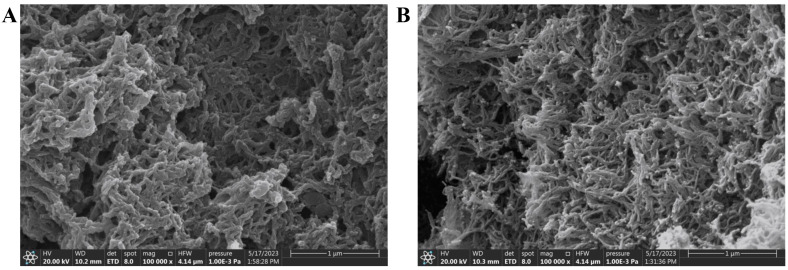
The SEM images of TpDMB-COPs-MMM (**A**) and TpDMB-COPs powder (**B**) (Magnification: 100,000×).

The TpDMB-COPs was characterized using TGA, BET, and SEM. The TGA curve shown in Figure S2 indicated that the TpDMB-COPs possessed high thermal stability and could be stable at 412 °C. The N_2_ adsorption isotherm of TpDMB-COPs was illustrated in Figure S3. The TpDMB-COPs display a representative type-IV isotherm at relative 0.3–1.0 pressure, thereby suggesting the existence of porous construction and the BET surface area was 142 m^2^/g^−1^. The surface morphology of synthesized TpDMB-COPs was evaluated using the SEM shown in Figure 1B. The high magnification SEM image of TpDMB-COPs revealed a porous network and nanoskeleton structure, presenting the successful synthesis of TpDMB-COPs.

The TpDMB-COPs-MMM was also characterized through SEM. The SEM image was presented in Figure 1A, showing a clear powder morphology on the surface, which demonstrated successful preparation of TpDMB-COPs-MMM while maintaining the powder’s morphology and properties. The SEM also indicated that the TpDMB-COPs were well-dispersed on the TpDMB-COPs-MMM surface, leading to an increased roughness of its surface. Furthermore, due to the hydrophobic nature of TpDMB-COPs, when it was added to PVDF to prepare a mixed matrix membrane, might cause an increase in the hydrophobicity of TpDMB-COPs-MMM. This can be confirmed by the contact angle measurement.

**Figure 2:**
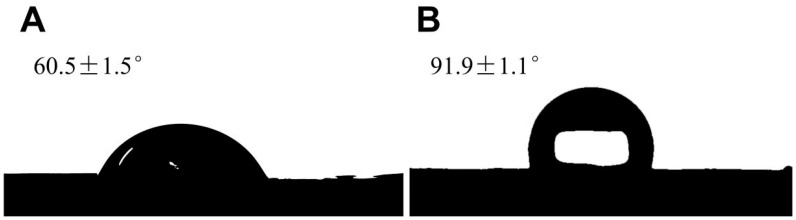
The static water contact angles of (**A**) pristine bare membrane and (**B**) TpDMB-COPs-MMM.

The contact angle measurements were conducted at room temperature using the sessile drop method. The results indicated an increase in the TpDMB-COPs-MMM contact angle (Figure 2B) from 60.5 ± 1.5° to 91.9 ± 1.1° compared with the pristine bare membrane (Figure 2A). This increase can be attributed to the increased surface roughness and hydrophobicity after treatment with TpDMB-COPs [20]. The surface contact angle of TpDMB-COPs-MMM was increased due to the hydrophobic nature of the carbon-carbon double bond skeleton of TpDMB-COPs. These results confirmed the successful synthesis of TpDMB-COPs-MMM [38].

### 2.2. Optimization of ME Procedures

To enhance extraction efficiency, we investigated several key factors, including pH, salt concentration, extraction time, type of eluent, eluent volume, and elution time, using the recoveries of the six SAs varieties as an evaluation indicator.

#### 2.2.1. Effect of the Sample pH

**Figure 3:**
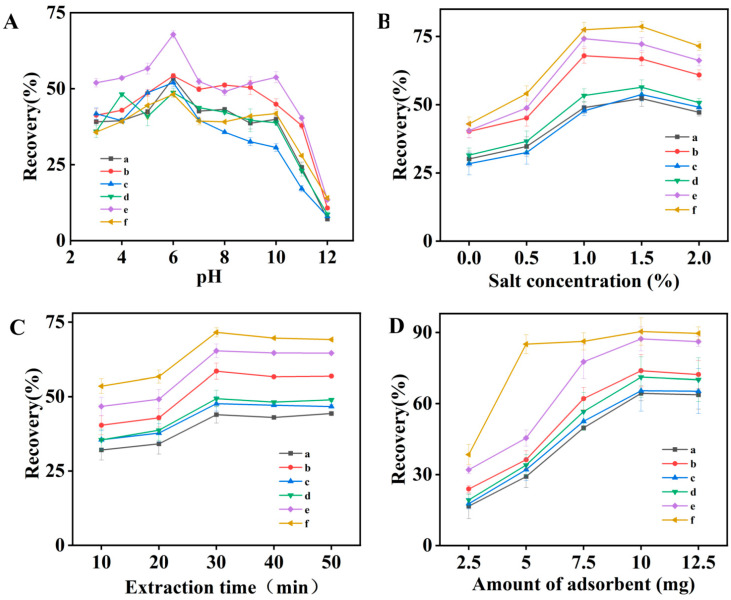
The investigation of sample pH (**A**); salt concentration (**B**); amount of adsorbent (**C**), and extraction time (**D**) (a: SMR, b: SMP, c: SMX, d: SDX, e: SDM, and f: SQ).

The pH of the extraction solution plays a crucial role in shaping the molecular or ionic states of SAs, in addition to affecting their interaction with the absorber (TpDMB-COPs-MMM). Thus, optimizing the pH was the first parameter studied. The effect of pH on extraction efficiency was investigated across various pH values ranging from 3.0 to 12.0. Figure 3A indicated that recoveries increased as the pH increased from 3.0 to 6.0, with the highest recoveries achieved at pH 6.0, and for most SAs, the recoveries decreased with further increases in pH from 6.0 to 10.0, though increased again at pH 10.0 for SDM and SQ. Nevertheless, for SDM and SQ, the recoveries at pH 10.0 remained lower than those at pH 6.0. And a significant decrease in the recoveries was observed when pH exceeded 10.0. This can be attributed to the amphoteric nature of SAs, where different pH levels dictated the prevalence of ionized and molecular forms [39,40,41]. They might exist in an ionic state at pH values below 6.0 or above 10.0, and be molecular states at 6.0 and 10.0 which are more likely to be adsorbed due to the hydrophobic force. Consequently, a sample pH of 6.0 was selected for subsequent analyses.

#### 2.2.2. Effect of the Salt Concentration

The impact of salt concentration on extraction efficiency is primarily observed in the salt effect. In order to investigate the influence of ionic strength on SAs extraction, different concentrations of NaCl were examined, with added NaCl ranging from 0% to 2% (*w*/*v*) (Figure 3B). For most SAs, the highest recoveries were achieved when 1.5% NaCl (*w*/*v*) was added to the sample. The extraction performance did not improve with further additional NaCl, probably due to increased sample viscosity. While the recoveries of SMP and SDM were the highest when adding 1.0% NaCl (*w*/*v*); however, the recoveries were not significantly different from those when 1.5% NaCl (*w*/*v*) was added. Therefore, subsequent experiments were conducted by adding 1.5% (*w*/*v*) NaCl to the sample solution.

#### 2.2.3. Effect of the Extraction Time

Extraction time refers to the duration required for the analytes to be adsorbed from the sample solution onto the sorbent. As the adsorption process followed an extraction procedure based on equilibrium, determining the optimal extraction time could guarantee efficient equilibration of analytes between the adsorbent and the aqueous phase. Figure 3C demonstrated that the extraction equilibrium was attained within roughly 30 min under continuous vortexing. Notably, the recoveries of most targets decreased with prolonged adsorption time. Hence, a 30 min extraction time was considered to be the optimal.

#### 2.2.4. Effect of Adsorbent Amount

The effect of the adsorbent amount on the recovery of six spiked SAs was investigated within the range of 2.5 to 12.5 mg. As displayed in Figure 3D, the highest recoveries was obtained when the amount of adsorbent was 10.0 mg, and the recoveries did not increase significantly when the amount of adsorbent exceeded 10.0 mg. It was concluded that the 10.0 mg of adsorbent was considered sufficient for subsequent experiments. The results also suggested that the developed sorbent exhibited high adsorption efficiency and could achieve extraction efficacy with a small amount of adsorbent, such that 10.0 mg of adsorbent was established as the optimal resin amount for subsequent experiments.

#### 2.2.5. Effect of Eluent Type and Volume

**Figure 4:**
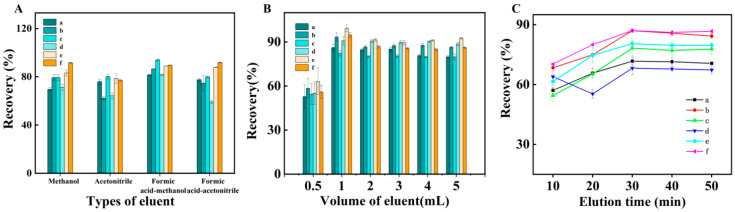
The investigation of types of eluent (**A**), volume of eluent (**B**), and elution time (**C**) (a: SMR, b: SMP, c: SMX, d: SDX, e: SDM, and f: SQ).

Since the adsorption of SAs onto TpDMB-COPs-MMM relied on π-π interactions, hydrogen bond forces, and hydrophobic interactions, it was necessary to consider desorption solvents with different polarities. Four eluents were examined, namely methanol, formic acid-methanol (1:1000, *v*/*v*), acetonitrile, and formic acid-acetonitrile (1:1000, *v*/*v*). Figure 4A illustrated that formic acid-methanol (1:1000, *v*/*v*) resulted in higher recoveries, making it the optimal eluent. Furthermore, the elution volume within the 0.5–5.0 mL was analyzed. The results (Figure 4B) revealed that the elution volume gradually increased with the volume of formic acid-methanol (1:1000, *v*/*v*) until reaching 1.0 mL. Further increases in volume beyond 1.0 mL did not lead to higher recoveries of the six SAs. Hence, a volume of 1.0 mL of formic acid-methanol (1:1000, *v*/*v*) was selected as the appropriate eluent.

#### 2.2.6. Effect of Elution Time

An appropriate elution time is crucial for ensuring the complete elution of an analyte and to maximizing extraction efficiency. In order to determine the optimal elution time, the effects of elution time between 10 and 50 min were studied. As depicted in Figure 4C, the recoveries increased rapidly between 10 and 30 min. However, when the elution time was further prolonged, the recoveries decreased slightly. This could be attributed to the equilibrium-based nature of the elution process, where over time, the targets might reattach to the adsorbent, resulting in a slight decrease in the elution efficiency. Thus, the elution time was fixed at 30 min.

#### 2.2.7. Reusability of TpDMB-COPs-MMM

Under optimal extraction conditions, the reusability of TpDMB-COPs-MMM as an adsorbent was assessed through five adsorption and desorption cycles. TpDMB-COPs-MMM was used as an adsorbent for a blank sample spiked with six SAs at concentration of 10 ng/mL. After completing the whole adsorption and desorption process, the TpDMB-COPs-MMM was taken out and washed with water and methanol. Then, it was put into spiked sample solution again for adsorption and desorption. As illustrated in Figure S4, the recoveries were slightly decreased after the fifth cycle. This indicated that the prepared TpDMB-COPs-MMM could be reused for at least five cycles.

#### 2.2.8. Comparison of Adsorption Capacity of TpDMB-COPs-MMM and TpDMB-COPs Powder

**Figure 5:**
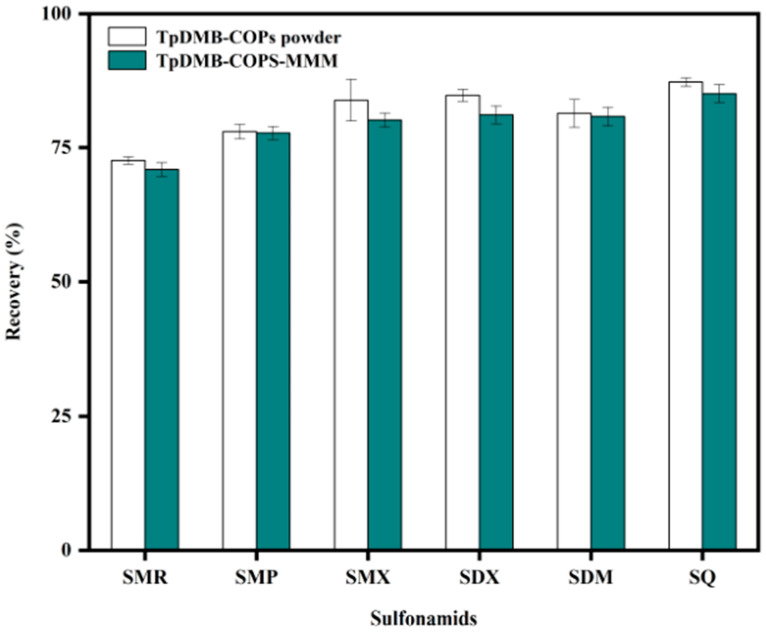
The comparison of adsorption performance of TpDMB-COPs-MMM material and TpDMB-COPs powder.

Under the optimal extraction conditions, the adsorption capacity of TpDMB-COPs-MMM was compared with TpDMB-COPs powder of the same mass. Figure 5 demonstrates that there is no significant difference in adsorption capacity between the TpDMB-COPs powder and TpDMB-COPs-MMM for six SAs. This finding provided evidence that the extraction performance of membrane was comparable to that of the powder. It indicated that converting the powder into a membrane did not change its adsorption ability and may even offer added convenience by eliminating the need for high-speed centrifugation for solid-liquid separation, and reducing adsorbent loss. Therefore, TpDMB-COPs-MMM was selected for detecting SAs in human urine.

### 2.3. Method Validation and Application to Real Samples

The developed ME-HPLC methodology was evaluated for linearity, the limits of detection (LOD), repeatability, the limits of quantification (LOQ), precision, and accuracy under optimized conditions. With pure water added standard mixed solutions of the six compounds at different concentrations were prepared as mock water samples at five concentrations levels within a specified range. These samples were then extracted under the optimized ME conditions, and analyzed by HPLC. The obtained working curves for the ME-HPLC extraction method yielded correlation coefficients (*r*^2^) ranging from 0.9991 to 0.9998. The detection and quantification limits for the six SAs ranged from 1.25 to 2.5 ng/mL and 3.5 to 7.0 ng/mL, respectively, with signal-to-noise ratios of 3 and 10. The results are presented in Table 1.

The precision of the method was evaluated based on intra-day and inter-day experiments, determined by assessing the relative standard deviation (RSD). Six replicates of spiked samples were applied to analyze the RSD on the same day, and three replicates of spiked samples were analyzed on three consecutive days. Detailed precision data can be found in Table S2. Accuracy experiments were conducted by spiking three concentrations of six SAs standard samples into blank human urine samples. The recoveries ranged from 87.4% to 112.2%, with RSDs less than 8.7%, demonstrating the reliability and applicability of the method. The results are presented in Table S3.

In order to assess the practicality of this approach, urine samples from volunteers were analyzed. The urine samples’ supernatant underwent pretreatment and were tested using the ME-HPLC method and the chromatograms were shown in Figure S4. Table 2 presents the results, indicating that SQ and SMR were detected in sample No. 2, with SQ at a concentration of 5.59 ng/mL and SMR below the LOQ. Sample No. 3 presented SMR at 17.65 ng/mL. In sample No. 4, SMR was detected at 14.39 ng/mL, and SQ was below the LOQ. Sample No. 6 indicated SMR at a concentration of 9.86 ng/mL. Sample No. 7 exhibited SDM at 19.82 ng/mL, while SMR and SMP were below the LOQ. SDM was detected in samples No. 7 but below the LOQ. No sulfonamides were detected in samples No. 1, No. 5, No. 8, and No. 10.

### 2.4. Comparison of the Developed Method with Previously Reported Methods

In order to highlight the application of this method in determining SAs in urine samples, a comparison with other techniques for SAs extraction was presented in Table 3. The developed method exhibited comparable or superior analytical performance in adsorbent amount and LOD compared with reported methods. Moreover, the developed TpDMB-COPs-MMM adsorbent used in this method was eco-friendly, low toxicity, and reusable. The comparison of analytical performance suggested that the membrane solid-phase extraction process with the TpDMB-COPs-MMM adsorbent was appropriate for the extraction and quantitative analysis of SAs in complex matrices.

## 3. Materials and Methods

### 3.1. Chemicals and Reagents

Six sulfonamide standards, including SMR, SMP, SMX, SDX, SDM, and SQ with purities of >98% were purchased from Aladdin Bio-Chem Technology Co., Ltd. (Shanghai, China), and their structures were illustrated in Table S1. Sodium chloride (NaCl), ethanol (EtOH), and acetone (ACE) were procured from Tianjin Kermel Chemical Reagent Co., Ltd. (Tianjin, China). PVDF and 2,4,6-triformylphloroglucinol (Tp) were purchased from Aladdin Bio-Chem Technology Co., Ltd. (Shanghai, China). p-Toluenesulfonic acid (PTSA) and 3,3′-dimethylbenzidine (DMB) were purchased from Xilong Scientific Co., Ltd. (Shantou, China). Dimethylformamide (DMF) was purchased from Beijing Tongguang Fine Chemical Company. (Beijing, China). All materials and reagents were of at least analytical grade and used as received. Methanol and acetonitrile with chromatographically pure were purchased from Shanghai Xingke Bio-Chem Technology Co., Ltd. (Shanghai, China). Ultra-pure water was obtained from a Thermo LabTower EDI 15 ultra-pure water system (Thermo Scientific, Stockholm, Sweden).

### 3.2. Instrumentation

The adsorbent’s morphological analysis under a scanning electron microscope (SEM) was performed using an Apreo S (Thermo Fisher Scientific, Waltham, MA, USA). Thermogravimetric analysis (TGA) was conducted by using TGA/DSC 1 analyzer (Mettler Toledo, Greifensee, Switzerland). The Brunauer-Emmett-Teller (BET) specific surface area was analyzed by using an ASAP 2010 apparatus (Micromeritics, Norcross, GA, USA). Analysis via chromatography was performed using an Agilent 1260 HPLC system with diode array detection (Agilent Technologies Co., Ltd., Santa Clara, CA, USA). The pH values were determined with a PHS-3C pH meter (Shanghai Leici Scientific Instrument Co., Ltd., Shanghai, China). For TpDMB-COPs-MMM preparation, an electronic hot plate (Shanghai Electronic Technology Co., Ltd., Shanghai, China), an ultrasonic cleaner (Shanghai Shangyi Instrument Equipment Co., Ltd., Shanghai, China), and a Vortex-2500 MT vortex mixer (Lichen Instrument Technology Co., Ltd., Shaoxing, China) were employed. Stock solutions were prepared in methanol and stored at −20 °C, while working solutions were prepared daily by diluting the stock solutions with ultrapure water.

### 3.3. Synthesis of TpDMB-COPs

In this study, the TpDMB-COPs were synthesized following a previous report by heating reflux [51]. Specifically, 0.021 g of Tp (0.1 mmol), 0.032 g of DMB (0.15 mmol), and 0.077 g of PTSA (0.45 mmol) were mixed with 150 mL of DMF and stirred for 12 h at 165 °C under N_2_ atmosphere. The resulting orange-red powder was collected after solvent exchange with DMF and EtOH, and subsequent overnight drying under vacuum.

### 3.4. Preparation of TpDMB-COPs-MMM

The TpDMB-COP powder (15 mg) was dispersed in ACE (2 mL) by sonication in a bath for 5 min, resulting in a TpDMB-COPs suspension. This suspension was subsequently mixed with a PVDF solution (0.6 g, 7.5 wt% in DMF) to achieve a final TpDMB-COPs to PVDF ratio of 1:3 (*w*/*w*). The mixture was agitated for 15 min at 65 °C on an electronic hot plate to remove ACE, obtaining a suspension of TpDMB-COPs and PVDF in DMF. The DMF suspension was evenly coated onto a glass substrate (1.8 cm × 1.8 cm), which was then heated to 65 °C on the plate to remove solvent. The coated films were delaminated from the glass substrate using a slide and stored. The preparation procedure was shown in Figure S1.

### 3.5. Sample Collection

Urine samples were collected from 10 volunteers. All human urine samples were filtered through a 0.22 μm membrane to remove suspended particles and then stored in brown glass vials at 4 °C in a refrigerator before analysis. Analysis of the samples was performed within 3 days.

### 3.6. ME Procedure

After activation with methanol and water in sequence, TpDMB-COPs-MMM was loaded into a 15 mL centrifuge tube containing 10 mL of sample solution with pre-adjusted pH and salinity. The mixture was vortexed for 30 min. Subsequently, TpDMB-COPs-MMM was easily removed with tweezers, dried with filter paper, and transferred to a 2 mL centrifuge tube. Precisely 1.0 mL of formic acid-methanol (1:1000, *v*/*v*) was used for eluting the target compounds from TpDMB-COPs-MMM by vigorous vortexing for 30 min. Finally, the TpDMB-COPs-MMM was removed, the eluent was filtered through a 0.22 μm filter membrane, and an aliquot of 10 μL was injected into the HPLC-DAD system for analysis.

### 3.7. HPLC Conditions

Chromatographic separation was performed using an Agilent 1260 HPLC system equipped with a DAD detector. An ODS-100V column (150 mm × 4.6 mm, 3 μm) was employed at 30 °C, with an injection volume of 10 μL. The detection wavelength was set at 268 nm. The mobile phase consisted of an aqueous solution of 0.1% formic acid (solvent A) and methanol (solvent B) at a flow rate of 1.0 mL/min. Prior to use, the mobile phase was filtered through a 0.22 µm filter. The system employed the following linear gradient: 0 min (72% A, 28% B), 8.0 min (72% A, 28% B), 12.0 min (62% A, 38% B), 17.0 min (52% A, 48% B), 19.0 min (72% A, 28% B), and 30.0 min (72% A, 28% B). All six compounds were successfully separated within 30 min.

## 4. Conclusions

In this study, a novel mixed matrix membrane named TpDMB-COPs-MMM was prepared by combining covalent organic framework polymers (TpDMB-COPs) with PVDF for the first time. TpDMB-COPs-MMM proved to be an effective sorbent for extracting SAs from human urine, followed by the HPLC analysis. The developed ME technology offered the advantage of simple operation and TpDMB-COPs-MMM can be easily separated from water without additional equipment, such as a centrifuge or vacuum pump. Notably, TpDMB-COPs-MMM possessed several advantages. It was cost-effective, easy to prepare, and can be reused at least 5 times without losing extraction efficiency, thus reducing total analysis time and cost. The method has a low detection limit, excellent precision, is accurate and reproducible, which makes it suitable for the detection of trace amounts of contamination in human urine samples. These properties make it a promising candidate for further development in the context of food safety and public health applications. Furthermore, the preparation of COPs-mixed matrix membrane also provided a potential avenue for the utilization of other COPs. This process served as a valuable reference for extending the application of COPs in sample preparation.

## Figures and Tables

**Table 1:** Linearity and range and results of LOD, LOQ.

Analytes	LOD (ng/mL)	LOQ (ng/mL)	Range (ng/mL)	*r* ^2^
SMR	1.25	3.50	3.5–20	0.9995
SMP	1.88	5.00	5–25	0.9998
SMX	2.50	7.00	7–25	0.9991
SDX	2.50	7.00	7–25	0.9997
SDM	2.50	7.00	7–25	0.9995
SQ	1.25	3.50	3.5–20	0.9993

**Table 2:** Determination of the target analytes in human urine.

Number	Concentration (ng/mL)					
	SMR	SMP	SMX	SDX	SDM	SQ
1	—	—	—	—	—	—
2	<LOQ	—	—	—	—	5.59
3	17.65	—	—	—	—	—
4	14.39	—	—	—	—	<LOQ
5	—	—	—	—	—	—
6	9.86	—	—	—	—	—
7	<LOQ	<LOQ	—	—	19.82	—
8	—	—	—	—	—	—
9	—	—	—	—	<LOQ	—
10	—	—	—	—	—	—

—: not detected.

**Table 3:** Comparison of the developed method with previously reported methods for determination of sulfonamides.

Extraction and Analytical Method	Analytes	Sample Matrix	Amount of Adsorbent	LOD	References
d-SPE/HPLC-DAD	SMR, SMP, SMX, SDM	Chicken, Lamb, Beef	15 mg	2.4~4.9 ng/g	[42]
MLC-DAD	SMR	Medicated feeds	—	32.7 mg/kg	[43]
LPME/HPLC	SMR, SMX	Urine	—	33~57 ng/mL	[44]
HPLC-MS/MS	SDX	Herbal products	—	0.002 μg/mL	[45]
MSPE/HPLC-DAD	SDM	Milk	50 mg	2.5 μg/kg	[46]
MSPE/HPLC–DAD	SMX, SDM, SQ	Milk	10 mg	12~14 μg/L	[47]
d-SPE/HPLC-FLD	SMX, SDM, SDX	Chicken, Muscle, Egg	300 mg	4.1~5.5 μg/kg	[48]
VUA-DLLMEDES/HPLC-PDA	SDM	Fish	0.6 mL	5.5 μg/kg	[49]
SPME/HPLC	SMP	Water	—	2 ng/mL	[50]
ME/HPLC-DAD	SMR, SMP, SMX, SDX, SDM, SQ	Urine	10 mg	1.25~2.50 ng/mL	This work

“—” stands for the relevant content not mentioned in the article; d-SPE: dispersive solid phase extraction; MLC: micellar liquid chromatography; LPME: liquid phase microextraction; MSPE: magnetic solid-phase extraction; VUA-DLLMEDES: vortexultrasonic assisted dispersive liquid-liquid microextraction in combination with hydrophobic deep eutectic solvents; SPME: solid phase microextraction; DAD: diode-array detector; LC-MS/MS: liquid chromatograph-tandem mass spectrometer; HPLC-FLD: high performance liquid chromatography-fluorescence detection; PDA: photo-diode array.

## Data Availability

The data presented in this study are available on request from the corresponding author.

## Supplementary Materials

The following supporting information can be downloaded at: https://www.mdpi.com/article/10.3390/molecules28217336/s1. Figure S1. The preparation procedure of TpDMB-COPs-MMM. Figure S2. The TGA curve of TpDMB-COPs powder. Figure S3. The N_2_ adsorption-desorption isotherms of TpDMB-COPs powder. Figure S4. Reusability of TpDMB-COPs-MMM. Figure S5. The chromatograms of spiked and real samples. Table S1. The chemical structure of six sulfonamides. Table S2. The results of precision. Table S3. The results of recoveries experiment.
