# Supplementary material for: Preparation of COPs Mixed Matrix Membrane for Sensitive Determination of Six Sulfonamides in Human Urine
Source: Molecules. 2023 Oct 30;28(21):7336. doi: 10.3390/molecules28217336 (PMC10649119; doi:10.3390/molecules28217336)
Supplement: Supplementary file 1 [file molecules-28-07336-s001.zip › molecules-2695462-supplementary.pdf]

Electronic Supplementary Information

Preparation of COPs Mixed Matrix Membrane for Sensitive Determination of Six Sulfonamides in Human Urine

Ying Liu [1], Yong Zhang [1], Jing Wang [2], Kexin Wang [1], Shuming Gao [1], Ruiqi Cui [1], Fubin Liu [1] and Guihua Gao [1,*]

[1] School of Pharmacy, Jining Medical University, Rizhao 276826, China; ying1737883492@126.com (Y.L.); zhang_2211@163.com (Y.Z.); tawkx589@163.com (K.W.); 17860202743@163.com (S.G.); 13697839528@163.com (R.C.); liufblvy@163.com (F.L.)

[2] School of Pharmacy, Shandong University of Traditional Chinese Medicine, Jinan 250355, China; wangjing20210903@163.com

* Correspondence: guihua526@163.com; Fax: +86-633-2983690

Content List

1. The preparation procedure of TpDMB-COPs-MMM (Figure S1), Page 3

2. The TGA curve of TpDMB-COPs powder (Figure S2), Page 4

3. The N_2 adsorption-desorption isotherms of TpDMB-COPs powder (Figure S3), Page 5

4. Reusability of TpDMB-COPs-MMM (Figure S4), Page 6

5. The chromatograms of spiked and real samples (Figure S5), Page 7

6. The chemical structure of six sulfonamides (Table S1), Page 8

7. The results of precision (Table S2), Page 9

8. The results of recoveries experiment (Table S3), Page 9

1. The preparation procedure of TpDMB-COPs-MMM

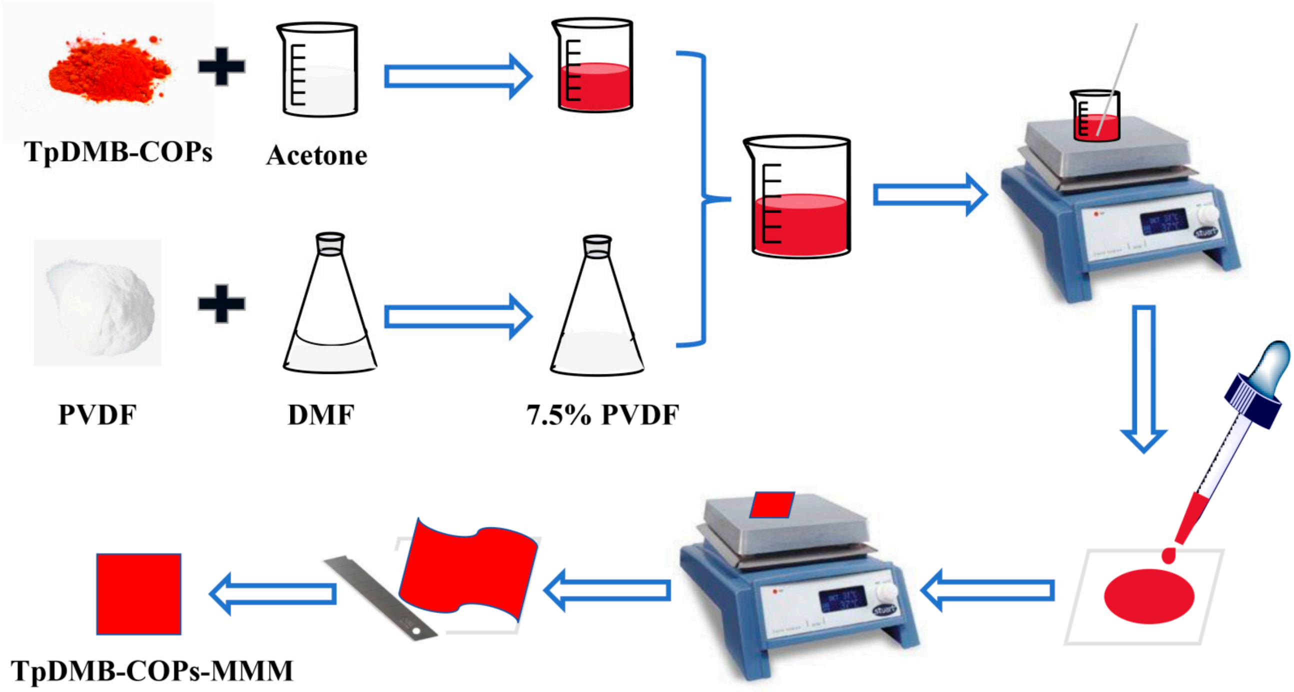

Figure S1 1. The preparation procedure of TpDMB-COPs-MMM.

2. The TGA curve of TpDMB-COPs powder

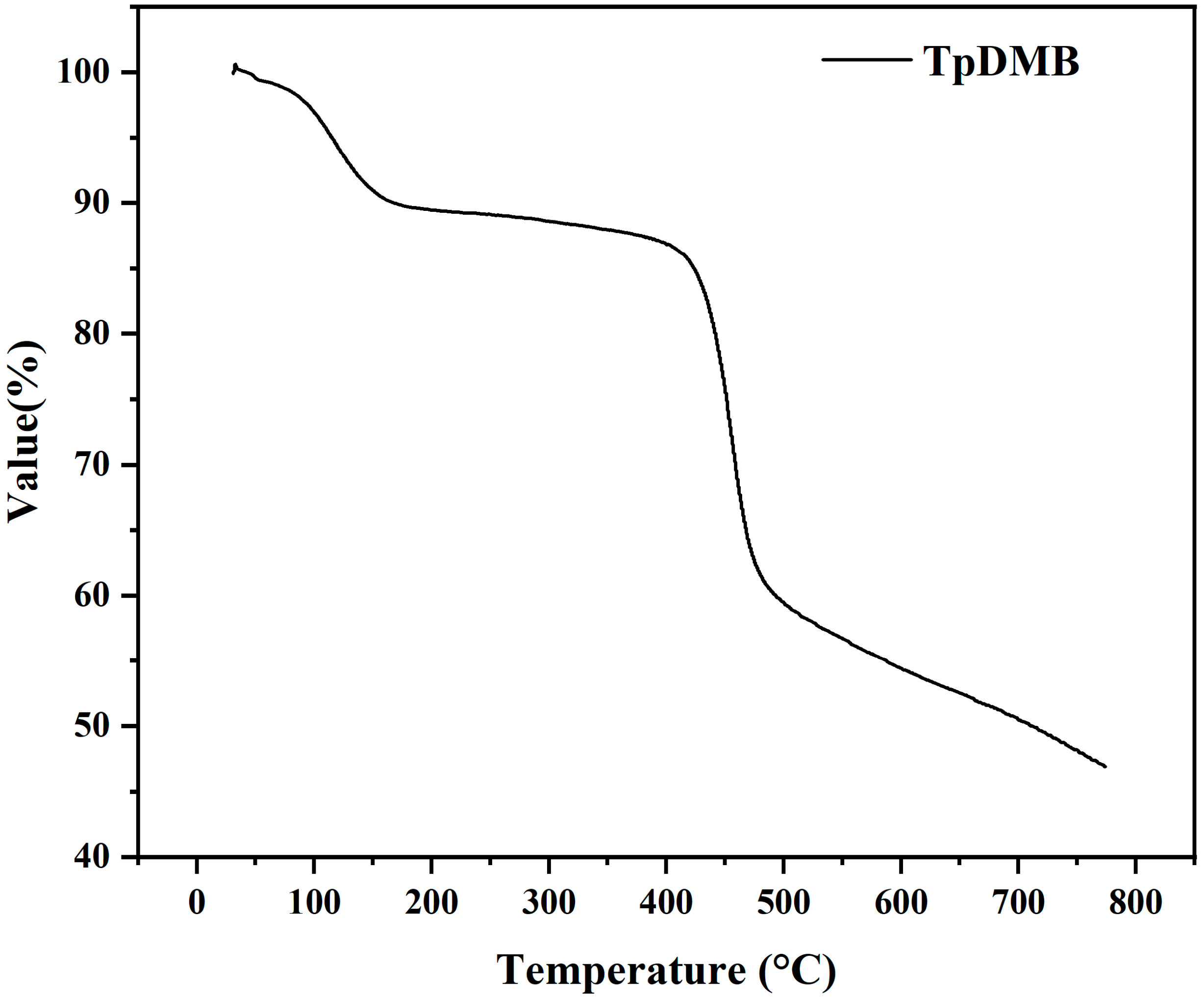

Figure S2 The TGA curve of TpDMB-COPs powder.

3. The N_2 adsorption-desorption isotherms of TpDMB-COPs powder

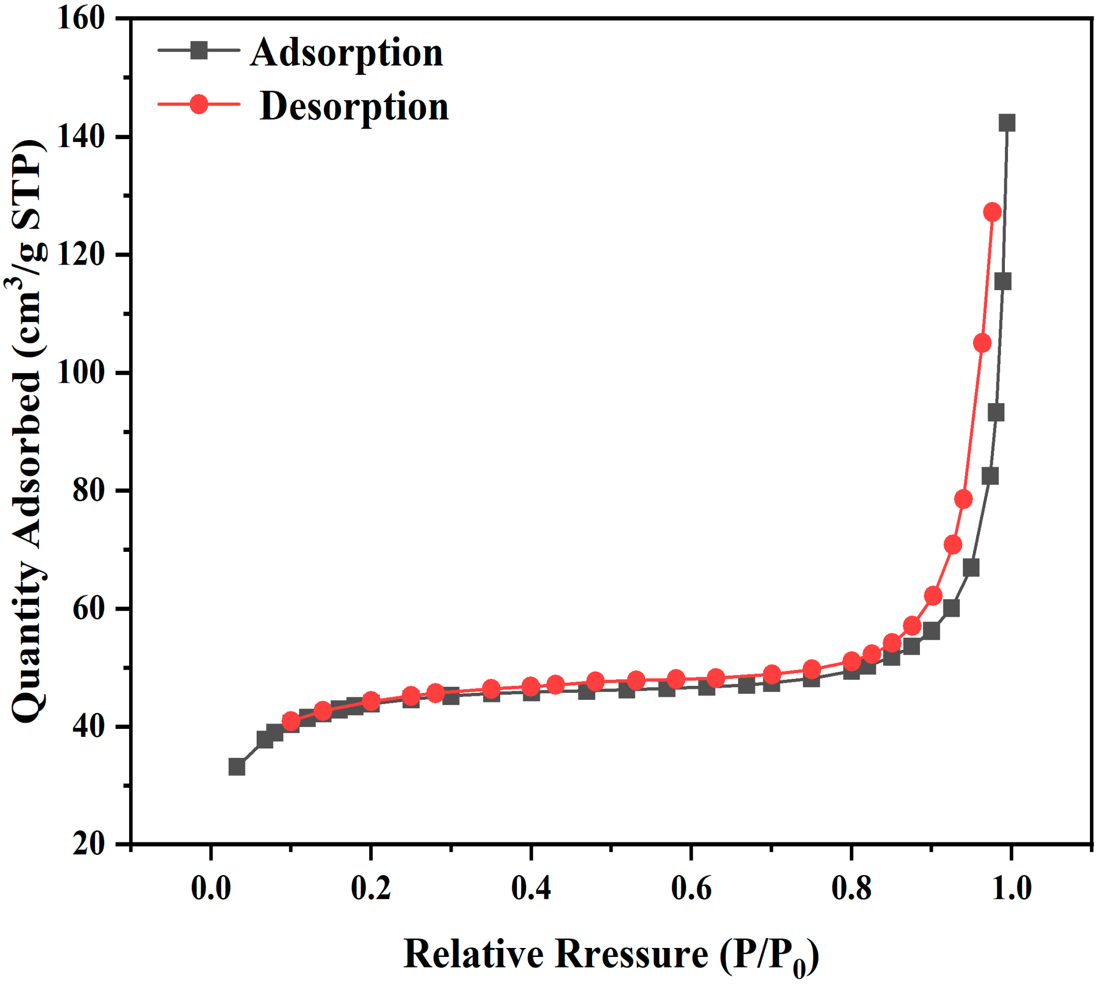

Figure S3 The N_2 adsorption-desorption isotherms of TpDMB-COPs powder.

4. The Reusability of TpDMB-COPs-MMM

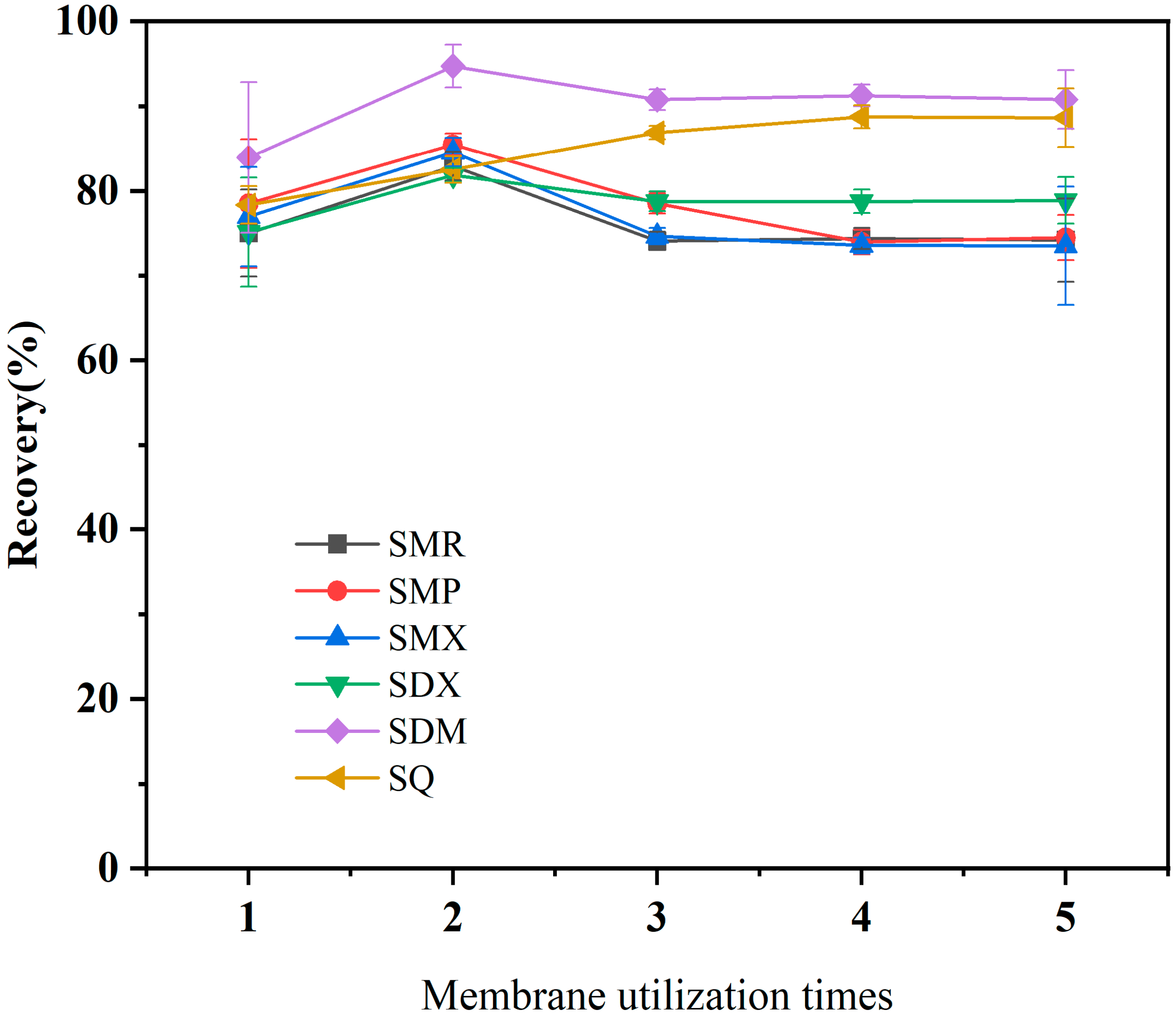

Figure S4 Reusability of TpDMB-COPs-MMM.

5. The chromatograms of spiked and real samples

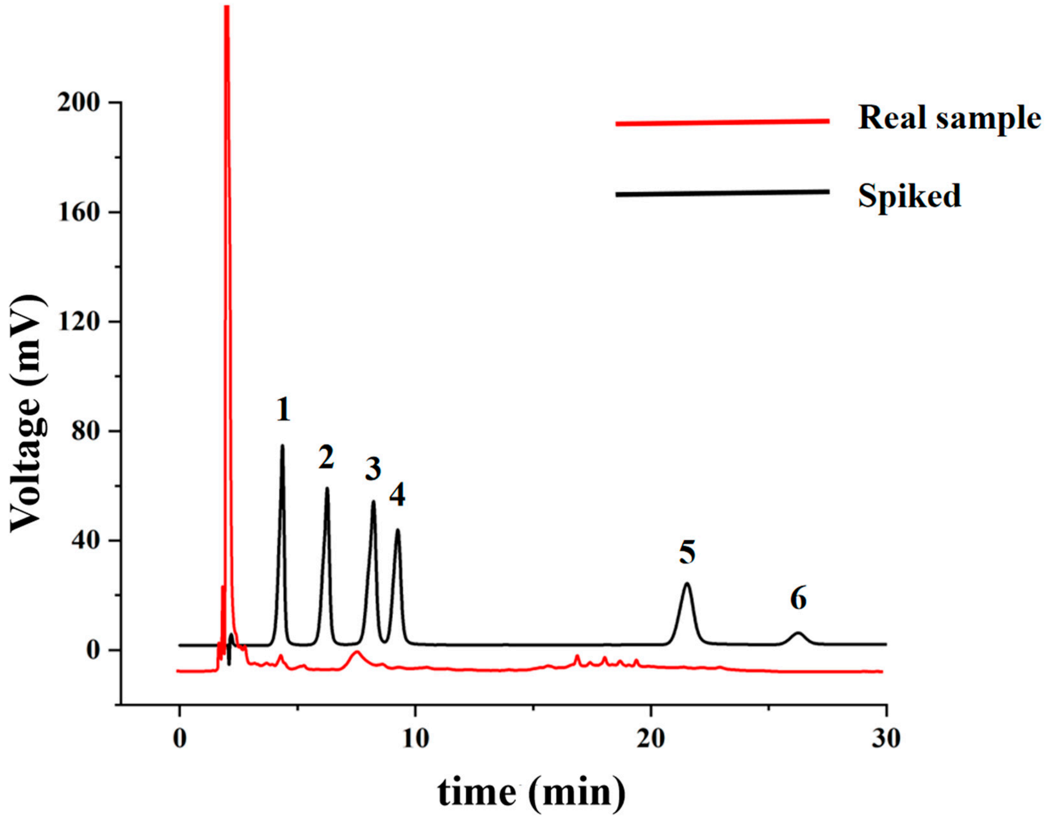

Figure S5 The chromatograms of spiked and real samples**.** Peak identification: 1, SMR; 2, SMP; 3, SMX; 4, SDX; 5, SDM; 6, SQ

6. The chemical structure of six sulfonamides

Table S1 The chemical structure of six sulfonamides

Sulfonamides	Chemical structure
Sulfamerazine (SMR)	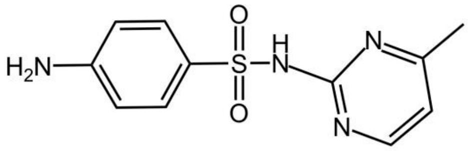
Sulfamethoxypyridazine (SMP)	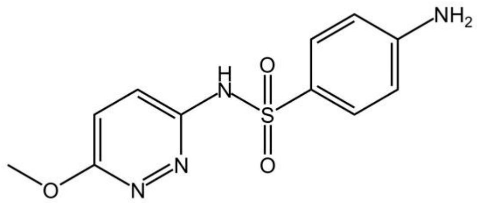
Sulfamethizole (SMX)	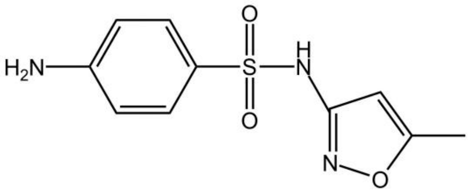
Sulfadimethoxine (SDM)	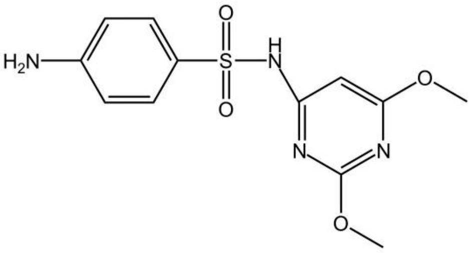
Sulfadoxine (SDX)	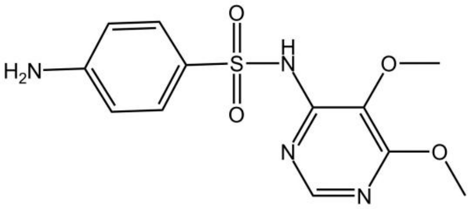
Sulfaquinoxaline (SQ)	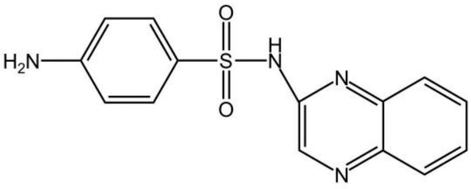

7. Results of precision

Table S2 Results of precision

Analyses	Repeatability (RSD%, n=6)	Intra-day (RSD%, n=6)	Inter-day (RSD%, n=9)
SMR	6.8	2.5	3.8
SMP	4.1	2.9	4.7
SMX	5.1	4.3	4.4
SDX	3.7	1.7	2.4
SDM	7.3	1.7	1.5
SQ	5.0	4.5	5.0

8. The results of recoveries experiment

Table S3 The results of recoveries experiment

Analytes	Low[a]		Middle[b]		High[c]	
	Repeatability （%）	RSD (%)	Repeatability （%）	RSD (%)	Repeatability （%）	RSD (%)
SMR	109.6	7.9	92.6	4.1	110.8	4.1
SMP	102.2	5.2	96.4	1.7	102.3	4.3
SMX	98.8	3.8	104.3	1.1	91.2	3.4
SDX	106.1	7.5	87.4	5.6	110.6	5.3
SDM	108.1	7.4	91.1	2.7	112.2	2.5
SQ	92.9	8.7	87.9	6.4	90.7	6.2

a: The low concentrations of SMR, SMP, SMX, SDX, SDM, SQ were 7 ng/mL, 10 ng/mL, 14 ng/mL, 14 ng/mL, 14 ng/mL, 7 ng/mL, respectively.

b: The middle concentrations of SMR, SMP, SMX, SDX, SDM, SQ were 10 ng/mL, 15 ng/mL, 15 ng/mL, 15 ng/mL, 15 ng/mL, 10 ng/mL, respectively.

c: The high concentrations of SMR, SMP, SMX, SDX, SDM, SQ were 16 ng/mL, 20 ng/mL, 20 ng/mL, 20 ng/mL, 20 ng/mL, 16 ng/mL, respectively.
